# Comparative Evaluation of Shear Bond Strength of Bioactive Restorative Material, Zirconia Reinforced Glass Ionomer Cement and Conventional Glass Ionomer Cement to the Dentinal Surface of Primary Molars: an *in vitro* Study

**DOI:** 10.30476/DENTJODS.2021.87115.1230

**Published:** 2021-12

**Authors:** Komal Nanavati, Farhin Katge, Vamsi Krishna Chimata, Debapriya Pradhan, Aishwarya Kamble, Devendra Patil

**Affiliations:** 1 Postgraduate Student, Dept. of Pediatric and Preventive Dentistry, Terna Dental College, Navi Mumbai, Indian; 2 Head of Dept. of Pediatric and Preventive Dentistry, Terna Dental College, Navi Mumbai, Indian; 3 Dept. of Pediatric and Preventive Dentistry, Terna Dental College, Navi Mumbai, Indian

**Keywords:** Glass ionomer cements, Composite resins, Zirconium, Dentin, Shear strength, Primary teeth

## Abstract

**Statement of the Problem:**

The success of dental restorations depends mainly on its ability to bond to dental structures and resist the multitude of forces acting on it within the oral cavity.

**Purpose:**

Therefore, the aim of this study was to evaluate the shear bond strength (SBS) of three different glass ionomer based restorative materials.

**Materials and Method:**

In this *in vitro* analytical study, 30 intact primary molars were sectioned buccolingually to obtain 60 sections. These sections were embedded in auto polymerizing acrylic resin and polished to obtain a flat dentin surface. Restoration cylinders were built on the dentin surface with the help of a Teflon template called bonding jig. Each group (n= 20) was restored as group A with conventional glass ionomer cement (GIC) (GC Fuji Gold Label Type 9), group B with Bioactive restorative material (ACTIVA^TM^ KIDS BioACTIVE Restorative material), and group C with Zirconia reinforced glass ionomer cement (Zirconomer). Following restoration, SBS testing was performed using Universal Testing Machine. The data obtained were statistically analyzed using One way ANOVA test and post hoc Tukey test (*p*= 0.05).

**Results:**

The SBS values were significantly greater in the ACTIVA KIDS group as compared to the other two groups (*p*< 0.05). There was no significant difference in the SBS values between group B and group C (*p*> 0.05).

**Conclusion:**

The SBS of the ACTIVA KIDS to primary teeth dentin was the highest as compared to Zirconomer and conventional GIC. Therefore ACTIVA KIDS may protect primary teeth against recurrent caries and failure of the restoration.

## Introduction

Caries is a common disease in children, for which the conventional treatment approach is to place a restorative material. For numerous years, dental amalgam has been the material of choice for restorations, but its application had several shortcomings. Its non-adhesive nature requiring additional depth and mechanical retention, unavoidable use of mercury and lack of esthetics led to a decrease in its popularity [ 1 ]. Thus, novel materials were introduced based on their adherence to tooth structure. This led to a revolution in cavity preparation wherein cavity size and shape was restricted to carious area [ 2 ]. Moreover, good marginal adaptation and bond strength are imperative for enhanced longevity of restorative material [ 3 ].

Wilson and Kent [ 4 ] introduced glass ionomer cement (GIC) in 1972. GIC is recommended in primary teeth, due to their ability to bond chemically to enamel and dentin. They have similar physical properties to the tooth structure. They present microleakage resistance and have ability to release fluoride ions over long periods [ 5 ]. However disadvantages like water sensitivity during initial setting period, long maturation time, low wear and abrasion resistance has eventually limited its use to areas where masticatory load is not high [ 6 ].

GIC has been modified several times to improve its physical and mechanical properties. The prerequisite for a more resilient material led to development of a new material with zirconia filler particles added to the glass ionomer composition [ 5 ]. This material is known as Zirconomer which is also referred to as “white amalgam.” Zirconia particles in zirconomer have strong optical and mechanical properties which provide the endurance of amalgam combined with fluoride discharging characteristics and biocompatibility of glass ionomer materials [ 7 ].

ACTIVA^TM^ KIDS BioACTIVE cement contains three key components: bioactive ionic resin matrix, shock-absorbing rubberized resin and reactive glass ionomer fillers. It contains many oxides that generate a strong bond with the tooth collagen by the production of hydroxyapatite. Bioactive resin of ACTIVA KIDS develops the natural remineralization process by continuously forming mineral apatite crystals, which form ionic bonds. This continuously forming bond reduces marginal gaps and microleakage which protects against recurrent caries and failure of the restoration [ 8 ].

Good adhesion of restorative materials with dentine surface increases its retention within the oral cavity. Shear bond strength (SBS) of a material resists forces that act obliquely on the restorative material. Consequently, higher SBS results in superior bonding between restorative material and tooth [ 9 ]. Thus, the aim of this study was to evaluate and compare the SBS of three different glass ionomer based restorative materials. The null hypothesis tested in this study was that there would be no significant difference between the three materials in terms of SBS. 

## Materials and Method

Sample size was determined in concordance to results from a previous study [ 4 ] through G^*^ power software (version 3.0.10). The total sample size calculated was 60 (20 per group). Protocol approval (number: TDCEC/ 10/2019) was attained from the Institutional review Board of Ethics for the current study.

### Preparation of samples

In the present study 30 primary molar teeth obtained from children aged between 7-10 years, with intact crown structure were included. The selected teeth were either extracted for orthodontic reasons or had exfoliated due to pre-shedding mobility. Teeth with fractured crown, any kind of developmental anomaly or caries were excluded to avoid related structural changes occurring in dentin due to these factors. Selected teeth were cleaned with a hand scaling instrument, following which they were examined under a light microscope at 20X magnification. The examined teeth were discarded if they had any visible structural defects, internal resorption, cracks or carious lesions. The teeth were then stored at room temperature in distilled water until use. The materials used in this study are presented in Table 1. The teeth were sectioned mesiodistally into buccal and lingual surfaces. A groove of 1.5mm depth from the enamel surface was created using a fissure diamond bur to assist in reaching a uniform depth of dentin in all samples. All sections were then embedded in auto polymerizing acrylic resin with either the buccal or lingual surface positioned for bonding with the restorative material. 

**Table 1:** The materials used in this study

Material name	Manufacturer	Composition
Conventional GIC-GC Fuji Gold Label Type 9	GC Co. Tokyo, Japan	Powder: silica, alumina, aluminium fluoride, calcium fluoride, sodium fluoride and aluminium phosphate.
		Liquid: polyacrylic acid
ACTIVA^TM^ KIDS BioACTIVE Restorative material	Pulpdent co., Massachusetts, USA.	Patented ionic resin
		Patented rubberized resin
		Bioactive glass ionomer
Zirconomer cement	Shofu Dental, Tokyo, Japan.	Powder: Fluoroaluminosilicate glass, zirconium oxide, pigment etc.
		Liquid: Polyacrylic acid, tartaric acid

After polymerization, the side of acrylic block with exposed enamel surface of tooth was ground in a polishing machine (Orien Dental Lathe Machine, Melbourne, Australia) using a silicon carbide paper of grit 600, under water cooling. All acrylic blocks with exposed enamel surface were standardized by polishing to a depth of 1.5mm in order to obtain an even layer of dentin. Exposed dentinal surfaces were evaluated with 20X magnification stereomicroscope (Motic Co. SMZ-143 series) to confirm that there was no remaining enamel or pulp chamber exposure after polishing [ 5 ]. 

### Restoration of samples

All the specimens were allotted randomly into three groups including Group A (n=20): conventional GIC, Group B (n=20): ACTIVA^TM^ KIDS BioACTIVE Restorative material, and Group C (n=20): Zirconomer.

An apparatus known as a jig with a Teflon template of height 2 millimeters (mm) and hole in the center of diameter 3mm was used. The inner walls of the hole were isolated with petroleum jelly to avoid sticking of restorative material each time the jig was used. The bonding jig was positioned for each sample in such a way that the hole was perpendicular to the exposed dentinal surface of the tooth. The jig was then tightened with a screw and bolt mechanism to receive restoration. 

 In the Group A( conventional GIC), conditioning of exposed dentinal surface was carried out with cotton pellet using GC dentin conditioner (GC Co. Tokyo, Japan) for 20 seconds. The surface was rinsed thoroughly with water and then blotted with a cotton pellet to remove the moisture. Powder and liquid were hand mixed in a ratio of 1:1 conforming to manufacturer’s instructions. Cement was then condensed onto the exposed dentinal surface through the hole of the jig. After setting of cement, the bonding jig was removed leaving behind a cylindrical extension of cement (height- 2mm diameter- 3mm) bonded to the dentinal surface. The cement surface was coated with GC Fuji COAT LC (GC Co. Tokyo, Japan).

**Figure 1:**
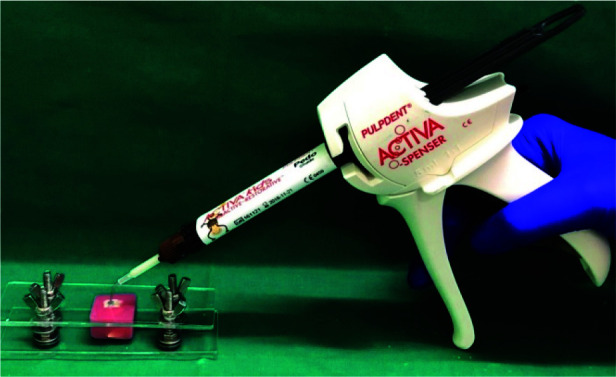
Dispensing ACTIVA KIDS using a spencer gun through the bonding jig template

**Figure 2:**
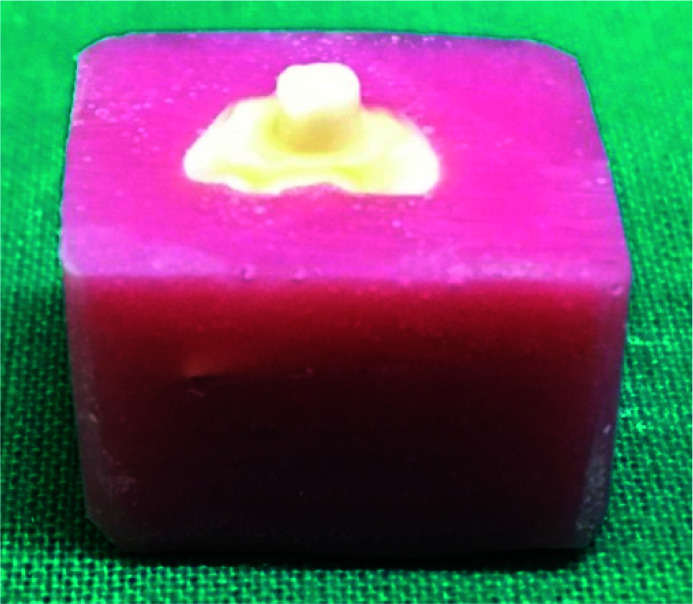
Sample with restorative cement built on to the dentin surface

In the Group B (ACTIVA^TM^ KIDS BioACTIVE Restorative material), the etching of the specimens was carried out using 37% phosphoric acid for 10 seconds followed by rinsing with water and air drying. SDI bonding agent (SDI Ltd. Victoria, Australia) was then applied and light cured using Ivoclar Vivadent Bluephase N M Light Cure Unit (New York, USA) for 20 seconds. Bonding jig was attached and ACTIVA KIDS was injected into the hole of the template using a spencer gun (Figure 1). Light curing of the specimens was carried out for 20 seconds, and then the bonding jig was removed and sample was obtained as stated above (Figure 2). The exposed ACTIVA KIDS surface was covered with glycerin (oxygen barrier) for its self-curing process.

In the Group C (Zirconomer), a powder to liquid ratio of 2: 1 was used as per manufacturer’s instructions. The cement was hand mixed and inserted onto dentin surface through the hole of the template. After setting of the cement, the bonding jig was removed. The surface was coated with cocoa butter (petroleum jelly) for protection against moisture. The restored specimens of all groups were stored in distilled water at 37°C for 24 hours.

### Evaluation of shear bond strength

**Figure 3:**
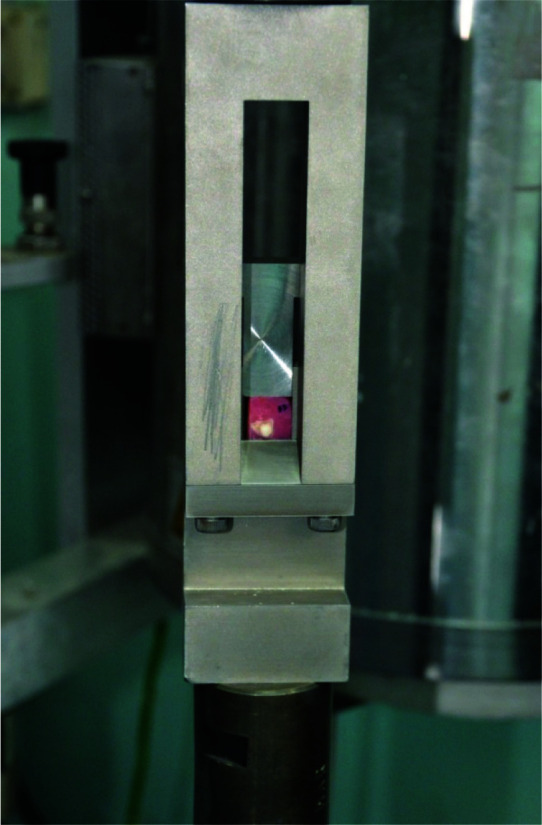
Sample with the cement mounted in universal testing machine for shear bond strength (SBS) evaluation

Universal Testing Machine (Zwick Roell) was employed to assess SBS. Each sample was placed and fastened in the Universal Testing Machine so as to keep the dentin surface parallel to machine’s trajectory. A steel knife-edge at speed 0.5 mm/minute was used to produce a shearing force at the bond interface between the sample and restorative cement (Figure 3). The maximum load necessary to cause debonding was recorded in a computer in Newton (N), and converted to megaPascal (a ratio of load to the surface area of cement). The sample surfaces were examined under a stereomicroscope with 10X magnification for fracture mode analysis [ 2 ].

Fracture modes were classified as adhesive (between the cement and dentin), cohesive (within the cement), or mixed (adhesive and cohesive fractures formed at the same time) [ 5 ]. The results were presented as percentages.

### Statistical analysis

One way ANOVA was the test used to analyze the data with *p*< 0.05 set as level of significance. Post hoc Tukey’s HSD test was performed to compare the scores between two groups. SPSS^®^ software version 17 was used to perform statistical analysis.

## Results

Each group (n= 20) was tested for SBS. The equation: 

Stress (MPa)= Failure load (N)/ surface area (mm^2^) was used to calculate the SBS values for each sample. The mean SBS was calculated for each group. 

Table 2 illustrates the mean SBS and standard deviation of conventional GIC, ACTIVA KIDS and Zirconomer. Group B ACTIVA KIDS showed the highest mean SBS value (6.4064) followed by Group C (3.8879) and Group A (2.3600) respectively, with the results being statistically significant (*p*< 0.05).

**Table 2:** Mean shear bond strength values and standard deviation of the three groups

	N	Mean	Standard deviation	STD Error	*p* Value
Glass ionomer cement	20	2.3600	1.00960	0.26983	
ACTIVA KIDS	20	6.4064	3.70013	0.98890	0.001
Zirconomer	20	3.8879	2.08639	0.55761	
Total	60	4.2181	2.98258	0.46022	

Significance level: *p* < 0.05

Inter-comparison between the three groups by applying Post hoc Tukey's test is depicted in Table 3. SBS values were found to have a significant difference between ACTIVA KIDS and conventional GIC as well as Zirconomer (*p*< 0.05) and the difference was not statistically significant (*p*> 0.05).

**Table 3:** Comparison of shear bond strength values between the three groups

Groups	Glass IonomerCement	ACTIVA KIDS	Zirconomer
Glass ionomer cement	-	0.001	0.256
ACTIVA KIDS	0.001	-	0.031
Zirconomer	0.256	0.031	-

Significance level: *p* < 0.05

According to failure mode analysis, Zirconomer (55%) and conventional GIC (60%) showed mostly mixed failure, while ACTIVA KIDS (90%) showed mostly cohesive failure. The failure modes for all groups (in percentage) are shown in Table 4.

**Table 4:** The following table shows the percentage for different types of failure modes in the three groups

Groups	Adhesive Failure	Cohesive Failure	Mixed Failure
Glass ionomer cement	4 (20%)	4 (20%)	12 (60%)
ACTIVA KIDS	1 (5%)	18 (90%)	1 (5%)
Zirconomer	5 (25%)	4 (20%)	11 (55%)

## Discussion

An ideal restorative material should have properties of good marginal adaptation, biocompatibility, chemical adhesion, and similar thermal expansion coefficient as the tooth. Dentin adhesion is a beneficial property as it can prevent the formation of secondary caries, microleakage, marginal discoloration, and subsequent pulpal damage [ 10 ]. Though glass ionomer chemically adheres to the tooth structure; it is not indicated in cavities wherein proper isolation cannot be achieved or in regions with high masticatory load [ 2 ].

**Figure 4:**
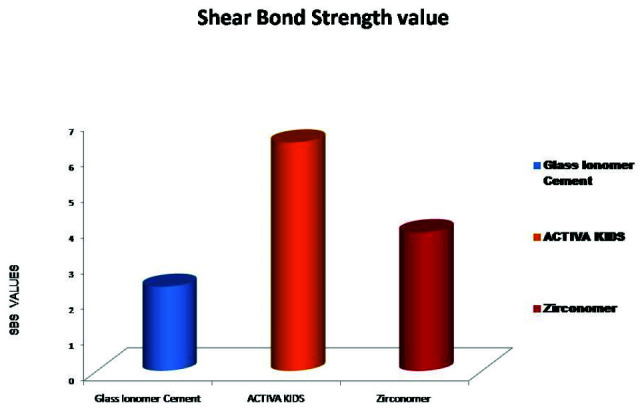
Distribution of shear bond strength values for all three groups

Various mechanical tests have been recommended for assessment of the bonding performance of restorative materials [ 11 ]. SBS testing is an important clinical property, since the majority of dislodging forces have a shearing effect at the tooth restoration interface [ 12 ]. In the present study, ACTIVA KIDS had the highest mean SBS value followed by Zirconomer and conventional GIC. Therefore, the null hypothesis was rejected (Table 2, Figure 4).

According to previous studies, the SBS of GIC to dentin is in the range of 1–3 MPa, rarely surpassing 5 MPa [ 3 , 9 , 13 ]. In a recent study Somani *et al*. [ 9 ] evaluated the SBS values of different types of GIC to primary tooth dentin. The SBS value was highest for light cure GIC, followed by type IX GIC; it was least for conventional GIC [ 9 ]. Similar values were found in a study by Almuammar *et al*. [ 14 ] wherein the mean SBS of conventional GIC was 3.77±1.76 MPa. This is in conformation with the current study where the SBS of GIC was 2.36 MPa. Conventional GIC forms an acid-base reaction between basic fluoroaluminosilicate glass powder and polycarboxylic acid [ 12 ]. Low SBS values observed in this group, may be due to inferior mechanical properties like low fracture toughness, wear resistance, tensile strength, and brittleness as compared to resin based GIC [ 15 ].

ACTIVA KIDS contains ionic resin and bioactive glass ionomer. The hydrophilic properties are exhibited by bioactive particles, where it extracts fluoride, calcium and phosphate from the saliva and releases these ions to the tooth. ACTIVA KIDS demonstrates intimate adaptation to the tooth structure. The chemical bonding that takes place between the tooth and the material creates durability and fracture resistance [ 16 ]. It therefore shows resemblance both to the physical qualities of GIC and traditional composite resin chemistry. A study conducted by Afutu *et al*. [ 8 ] reported higher SBS of ACTIVA KIDS to dentin as compared to GIC (Fuji IX GP Extra). The better performance of ACTIVA restorative material was attributed to its adhesion mechanism and improved mechanical characteristics [ 8 ]. Alkhudhairy *et al*. [ 17 ] compared the SBS of ACTIVA restorative with other bulk-fill restorative cements SureFil SDR, Biodentine, ever X posterior. The mean SBS for ACTIVA restorative, 6.28±0.157 MPa was similar to the value attained in the current study [ 17 ].

ACTIVA Bioactive Restorative has a resilient resin matrix that does not chip, resulting in significantly better physical properties and fracture resistance. ACTIVA restorative cement is composed of silica particles and polyacid components similar to resin-modified GIC, which will go through acid/base reaction as seen in GIC. In addition, the bioactive ionic resin matrix, which is a component of ACTIVA polymerizes by light cure and chemical cure [ 18 ]. Thus, these three setting mechanisms make ACTIVA restorative unique by incorporating physical properties analogous to those of the resin-based composites and biological characteristics similar to GIC [ 19 ]. ACTIVA stimulates the remineralization process by forming mineral apatite crystals. The bond thus formed is responsible for reducing marginal gaps and protecting the teeth against recurrent caries and failure of the restoration [ 3 ]. Therefore the improved properties of ACTIVA restorative material may contribute to the higher bond strength values as shown in the current study. 

Zirconomer is a Zirconia reinforced glass ionomer material marketed with the ability to eliminate the esthetic and mechanical disadvantages of conventional GIC [ 20 ]. In a study by Meral *et al*. [ 5 ], the SBS value of Zirconomer was greater than conventional GIC but the results were not statistically significant. In another in vitro study [ 21 ], Zirconomer was compared with conventional GIC and amalgam in terms of compressive strength. Zirconomer and amalgam showed similar compressive strength values, much greater than conventional GIC. The addition of zirconia as filler particles in the glass component of Zirconomer improves the mechanical properties of the restoration by reinforcing structural integrity of the restoration in load- bearing areas [ 5 ].

According to the results of the current study; ACTIVA KIDS restorative material mainly showed cohesive fracture whereas, Zirconomer and conventional GIC showed mostly mixed fracture. Therefore, it can be inferred from the presence of cohesive and mixed failures that the interfacial bond strength in these restorative materials is more than inherent strength of the material [ 22 ]. ACTIVA combined the physical properties of both resin based composites and resin modified GIC. The rubberized resin molecule in ACTIVA absorbs stresses and dissipates forces. These factors help in increasing the facture resistance of ACTIVA [ 8 ]. 

In the current study, Zirconomer and conventional GIC did not show a significant difference for SBS values, and ACTIVA KIDS restorative material was significantly higher than Zirconomer and conventional GIC. Hence, based on the results of this study, ACTIVA KIDS BioACTIVE restorative material can hold a place in minimally invasive techniques involving posterior restorations in pediatric dentistry.

There are only a few published studies regarding Zirconomer and ACTIVA KIDS, and this study is unique for comparing these materials. Further in vitro and in vivo research is required to examine the performance of newer glass ionomer based materials, aiming at application of these materials with an increased sample size while mimicking the oral environment.

## Conclusion

ACTIVA KIDS exhibited higher SBS values as compared to Zirconomer and conventional GIC, which was statistically significant. The bioactivity of ACTIVA KIDS protects against recurrent caries and failure of the restoration, leading to an overall better longevity and durability. Zirconia reinforced GIC can be used as an alternative for conventional GIC. 

## Acknowledgement

This study was conducted in the Bhabha Atomic Research Centre. I would sincerely like to thank Mr. Shreenivas Puttagunta, Dr. R. N. Singh, Mrs. Arnomitra Chatterjee and Mr. Kumawat for their help and support. 

Financial support and sponsorship: This research did not receive any specific grant from funding agencies in the public, commercial, or not-for-profit sectors. Institutional Ethics Review Board approved the study (Terna Dental College Ethics Committee; Registration No: ECR/1221/Inst/MH/2019, Protocol approval number: TDCEC/10/2019).

## Conflict of Interest

There are no conflicts of interest.
